# The Challenging Road towards a Unified Animal Research Network in Europe

**DOI:** 10.1371/journal.pbio.1002157

**Published:** 2015-05-27

**Authors:** Emma Martinez-Sanchez, Kirk Leech

**Affiliations:** The European Animal Research Association, London, United Kingdom

## Abstract

Animal models are key in biomedical research as a proof of concept to study complex processes in a physiological context. Despite the small yet crucial role animals play in fundamental and applied research, the value of animal research is recurrently undermined. Lack of openness and transparency encourages misconceptions, which can have a dramatic negative impact on science and medicine. Research centres should use all available resources to ensure that relevant details about their use of animals in research are readily accessible. More concerted efforts by professional advocacy groups devoted to informing about the benefits of biomedical animal research are also crucial. The European Animal Research Association acts as an umbrella organisation providing support to national advocacy groups and coordinating actions in countries in which no advocacy group exists.

## Abstract 2

This Perspective maintains that research bodies in Europe are in need of authoritative and independent professional organizations that explain why and how animals are used in scientific research.

## Introduction

Recent opinion polls in the United Kingdom [1] have shown that although 64% of the public is supportive of the use of animals in medical research, a similar percentage feels uninformed about science and scientific research and developments. Lack of transparency and openness in many European research centres encourages misconceptions about animal research. Worryingly, misinformation has a negative impact both on fundamental research that would not be possible without animal models and on those who perform it. Although more coordinated policing and a collapse in public support for extremist activity have led to a Europe-wide reduction in criminal activity aimed at halting animal research, scientists involved in animal research are still targeted for their work (Box 1).

**Box 1. Animal Rights Activists’ Tactics**

Animal rights activist groups run well-funded national and international initiatives aimed at winning the support of the general public and decision makers for their campaigns to restrict and, ultimately, abolish animal research. In September, a joint British Union for Abolition of Vivisection (BUAV) and Soko-Tierschutz undercover investigation at the Max Planck Institute for Biological Cybernetics in Tübingen, Germany, resulted in a misleading 7-min film showing images of monkeys used to study the brain [2,3]. Another high-profile campaign in Germany has targeted neurobiologist Andreas Kreiter, whose research with non-human primates (NHP) at the University of Bremen ranges from investigations into new treatments for epilepsy to the improvement of the control mechanisms for prosthetic devices. Professor Kreiter was portrayed last April as “not quite human” in a full-page newspaper advertisement [4,5]. The advertisement was circulated by the group Tierversuchsgegner Bundesrepublik Deutschland (Opponents of Animal Experiments Federal Republic of Germany) in several national and regional newspapers including *Zeit*, *Frankfurter Allgemeinen*, *Tagesspiegel*, *Weser-Kurier*, and the *Bremer Nachrichten*. In January 2014, scientists involved in animal research at the University of Milan were labelled as “murderers” in graffiti and leaflets that included their photos and contact information [6]. In April 2013, the Italian group Fermare Green Hill (Stop Green Hill) occupied an animal facility in the same university and released mice and rabbits, damaging years of research [7].

Without reliable, authoritative communication from the biomedical sector, public understanding can be manipulated through “leaks” and “exposés” that do not accurately reflect either the rationale and need for the research or the ethical standards to which such research is held. Unopposed, these campaigns can be extremely effective in influencing public opinion and coordinating political opposition to animal research. A salient current example is the Stop Vivisection European Citizens’ Initiative. The European Citizens’ Initiative was introduced by the Lisbon Treaty [8] to encourage greater democratic involvement of citizens in European affairs. The initiative allows any citizen of the European Union (EU) to call on the European Commission to propose legislation on matters of EU competence, provided the cause is supported by a petition of at least one million signatories coming from seven member states or more. The current Stop Vivisection initiative, organised and funded by ten Italian animal activist groups, aims to abrogate Directive 2010/63/EU on the protection of animals used for scientific purposes [9]. It collected 1,150,000 certified signatures and was submitted to the Commission in March 2015. The initiative states that animal experimentation is a hazard to human health and to the environment and that it halts the development of non-animal alternative methods. The petitioners present Directive 2010/63 as a step back and propose instead the compulsory use of alternative methods wherever applicable. Despite these claims and demands, the scientific community recognises the Directive as the world’s most progressive and stringent framework for ensuring high animal welfare standards while encouraging the development of non-animal alternatives [10]. The Stop Vivisection initiative conspicuously fails to acknowledge the proven benefits for human and animal health that this research has provided to date.

## Communications Organisations in Europe

Research centres lack a pan-European organisation tasked with improving communication about the benefits of using animals in research and the reasons for their continued use. This situation has led to a dangerous lack of balance in the publicly available information on animal research. The lack of openness and transparency from research centres regarding their animal research activities prompts distrust and results in a one-sided narrative. Professional communications should ensure that all stakeholders, from research staff to the general public and legislators, are properly informed about the circumstances when animal research is necessary and authorised and the limits and protections that apply to each specific case.

In Germany, the Alliance of Science Organisations, a union of the most important public research institutes, had for some time suspended plans to create an independent advocacy organisation. These plans were promptly reopened following the incident in Tübingen (Box 1). Despite the discouraging situation in Germany, positive lessons can be learned from their neighbours. In Italy, support for biomedical research has risen after the creation of Pro-test Italia [11], a non-profit organisation for the defence of biomedical research, at the end of 2012. Ipsos MORI, the market research agency commissioned by the UK government to track public attitudes towards the use of animals in scientific research in Great Britain, released the results of their latest poll on Italian public opinion on animal testing in January 2014 [12]. The previous 2012 survey [13] found that only 33% of interviewees were supportive of experimenting on animals for medical research purposes. That figure rose to 49% in 2014. This swing in public opinion reflects an improvement in the information available about animal research, which likely came from the engagement of groups such as Pro-test Italia and the Mario Negri Institute [14]. The founder of the Mario Negri Institute, Professor Silvio Garattini, played a fundamental role in ensuring that the Italian public heard the viewpoint of researchers. In November 2013, he organised the “Io Sto Con La Ricerca” (“I’m With Research”) Convention [15], which attracted more than 400 participants and aimed at emphasising the importance of biomedical research to human health and the role of animal research within it.

Despite the potential effectiveness of initiatives by organisations such as the Mario Negri Institute and Pro-Test Italia in communicating the need for and benefits of animal research, there are still only a handful of such advocacy groups operating in European countries. In Switzerland, the Basel Declaration Society (BDS) strives to “further advance the implementation of ethical principles and to promote trust, transparency and communication on the sensitive topic of animals in research” [16]. The Stichting Informatie Dierproeven (SID) is committed to inform about experimental animals in the Netherlands [17]. GIRCOR (Groupe Interprofessionnel de Réflexion et de Communication sur la Recherche) was created in France in 1991 to “unite scientists and public and private research organisations in their work to support animal experimentation” [18]. The Research Defence Society founded in 1908 operated in the UK for a century before it merged with the Coalition for Medical Progress. Together they formed Understanding Animal Research (UAR), a sound non-profit organisation that aims to achieve “broad understanding of the humane use of animals in medical, veterinary, scientific and environmental research in the UK” [19].

These advocacy organisations can help to counter misinformation on the use of animals for medical or scientific purposes. For example, the Stop Vivisection European Citizens’ Initiative opposes Article 13 of European Directive 2010/63 (on the protection of animals used for scientific purposes), claiming that protection for animals contained in the legislation—which states that an animal cannot be used if a recognised alternative is available—is inadequate because the EU does not recognise enough alternative, non-animal scientific procedures. The implication here is that non-animal experimental alternatives exist but that vested interests prevent them from being certified by EU legislation. This is a persuasive argument for many people unless the reasons for the limitations on non-animal alternatives—the danger to human health of using methods that have not been fully validated by the Union Reference Laboratory—are carefully explained. If the explanation is not forthcoming, this type of misinformation can have adverse consequences. Failing to effectively engage audiences in understanding why we still need the humane use of animals in research could potentially result in a ban on all research on animals. Ceasing animal research activities will not only affect the development of new medicines but will hinder the development of non-animal alternative methods as well since information collected from animal models, among other sources, is required to design alternative models.

Another example of misinformation that advocacy organisations can help to counter concerns the role of animal breeders and supplier companies in the context of animal research. These two professional groups are often the target of activists who want to halt their activities to decrease the number of animals used for research. They are soft targets for propaganda campaigns because the general public is often unaware that these companies are as carefully regulated through European Directive 2010/63 as any research organisation that uses their animals. They must show that they are expert in meeting the needs of each species that they supply and have taken every reasonable measure to improve welfare and minimise any distress or suffering. Furthermore, the efforts to halt supplier and breeder activities will likely boost rather than reduce research animal numbers, as individual research centres become forced to create their own, less efficient, breeding programmes to fill the gap.

One shared interest of all animal research advocacy groups is to ensure that everybody is accurately informed about scientists’ commitment to the 3R principles. The 3R principles set out the accepted standards for humane experimentation on animals, first introduced by Russell and Burch in 1959 in their book *The Principles of Humane Experimental Technique* [20]. This book is a systematic study of laboratory techniques in their ethical aspect. Russell and Burch classify humane techniques under the headings of “replacement,” “reduction,” and “refinement,” widely known as the 3Rs. The 3Rs are endorsed and implemented by all responsible scientists and have become embedded in national and international legislation regulating the use of animals in scientific procedures. This legislation requires that, where possible, all researchers (1) avoid or replace the use of animals by employing alternatives such as human volunteers, cell lines, or computer modelling, (2) reduce or minimise the number of animals used per experiment by maximising the amount of information obtained in relation to the number of animals used, using statistical analysis or sharing databases, and (3) refine or minimise the pain, suffering, distress, or lasting harm that may be experienced by the animals by, for example, using appropriate anaesthetics and analgesics or training animals to avoid stress.

The 3R principles outlines the animal welfare standards that are enshrined in the European Directive 2010/63/EU; however, its successful implementation relies on the proactive and coordinated engagement of the multiple stakeholders. From breeding, housing, and handling of experimental animals to designing and reporting animal experiments (Box 2), all measures should be taken to ensure minimal pain is caused and to overall reduce and replace animals used for scientific purposes.

**Box 2. The ARRIVE Guidelines**

In 2010, the ARRIVE (Animal Research: Reporting in Vivo Experiments) guidelines were published in this journal to improve the reporting of animal research [21]. These guidelines have been endorsed by many publishers and are set to standardise the experimental approach of animal experiments, aiming at ensuring reproducibility, and avoid unnecessary animal use.

The accuracy of animal models for predicting adverse effects in humans is often called into question by campaigners, and it is true that in some circumstances animal models add only limited value, which should be explained within the context of the studies’ limitations. This is a major concern for academics, industry leaders, research funding organisations, and governmental authorities alike. More effective, predictive preclinical models could deliver significant scientific and economic benefits. The development of such models—whether they are animal models or, ideally, non-animal replacements—will depend on the active engagement and cooperation of a broad range of research sectors to create strategic synergies that combine cutting-edge scientific knowledge and technical know-how with the best modern understanding of animal behaviour and welfare.

Organisations like the National Centre for the Replacement, Refinement and Reduction of Animals in Research (NC3Rs) are pioneers in fostering such multistakeholder research alliances. Established in 2004, the NC3Rs is a UK-based scientific organisation seeking to accelerate the development and application of science and technology to replace, reduce, and refine the use of animals in scientific purposes [22]. They collaborate with national and international scientists and organisations in the life sciences to support 3Rs research and training as well as open innovation and commercialisation of 3Rs technologies. By funding cutting-edge research and multidisciplinary approaches, they have positioned the UK at the forefront of 3Rs efforts globally. NC3Rs-funded projects have resulted in the phasing out of the lethal single-dose acute toxicity test [23], the development of in vitro drug-testing platforms [24], and the replacement of higher animal species with rodent models in potency tests for the development of novel therapeutics [23].

Similar organisations devoted to driving evidence-based changes in practice, policy, and regulatory frameworks are active in Scandinavian countries [25–28], Switzerland [29], Germany [30], and the Netherlands [31]. Core to the strategy of these centres is to establish synergies between animal welfare organisations and academic and industrial partners, to share knowledge, and to promote the development and refinement of procedures and alternatives to animal research.

Despite the progress achieved to date in replacing, reducing, and refining animal research procedures, animal models remain central to progress in the life sciences and the development of new medicines. Alternative models, no matter how promising at first sight, must be validated and authorised before they can be implemented if current standards of safety and effectiveness are to be maintained. The research community should be clear about this process and about the reasons behind the continued use of humane animal models in scientific and medical research and make use of all available resources to ensure its arguments are heard.

The benefits gained through individual animal research advocacy organisations can be amplified if a collective, clear response to activist pressure can be coordinated in Europe. This is the purpose of the European Animal Research Association (EARA) [32]. EARA fosters the creation of a pan-European network that will help to coordinate local and national advocacy groups and to facilitate the establishment of new networks where needed. By supporting European advocacy groups, EARA aims to help ensure that the public and legislators are accurately informed and can unite in support of high-quality, relevant, and necessary biomedical research using animals.

EARA is a membership organisation and we encourage research institutions that share our concerns about the absence of an intelligent narrative to the public on the benefits of animal research to join us. For further information, please visit www.eara.eu.

## Abbreviations

ARRIVE: Animal Research: Reporting in Vivo Experiments
BDS: the Basel Declaration Society
BUAV: British Union for Abolition of Vivisection
EARA: European Animal Research Association
EU: European Union
GIRCOR: Groupe Interprofessionnel de Réflexion et de Communication sur la Recherche
NC3Rs: National Centre for the Replacement, Refinement and Reduction of Animals in Research
NHP: non-human primates
SID: Stichting Informatie Dierproeven
UAR: Understanding Animal Research
