# Correction: The Challenging Road towards a Unified Animal Research Network in Europe

**DOI:** 10.1371/journal.pbio.1002205

**Published:** 2015-07-06

**Authors:** Emma Martinez-Sanchez, Kirk Leech

The Funding and Competing Interests statements for this article did not contain all the relevant information. The corrected, full statements can be found here.

## Funding

EMS and KL are employed by the European Animal Research Association (EARA – http://eara.eu/home/), a not-for-profit membership organisation that advocates for the continued responsible use of animals in biomedical research. EARA (and EMS and KL’s employment) is funded via membership fees from public and private research organisations that rely for their activities on the humane use of animals in scientific research; or benefit, directly or indirectly, from this research. The funders had no role in study design, data collection and analysis, decision to publish, or preparation of the manuscript.

## Competing Interests

EMS is the PR and Communications Officer of the European Animal Research Association (EARA); KL is the Executive Director of EARA. EARA is a not-for-profit advocacy and communications organization, whose mission is to inform and educate the public and key stakeholders about the continuing need for, and the benefit of, the humane use of animals for scientific purposes. Through this mission, EARA aims to create and foster favourable conditions in Europe for research using animals so that important medical and scientific research can continue.

## References

[1] Martinez-Sanchez E, Leech K (2015) The Challenging Road towards a Unified Animal Research Network in Europe. PLoS Biol 13(5): e1002157 doi:10.1371/journal.pbio.1002157 26018997 10.1371/journal.pbio.1002157 PMC4446354

